# The frequency of gonorrheal and chlamydial infections in Zanjanian women in 2013-2014

**Published:** 2017-02

**Authors:** Behnaz Molaei, Farnaz Mohmmadian, Maryam Eftekhar, Robabeh Hatami, Atefe Tirkan, Mahsa Kiani

**Affiliations:** 1 *Department of Obstetrics and Gynecology, Zanjan University of Medical Sciences, Zanjan, Iran.*; 2 *Department of Obstetrics and Gynecology, Shahid Sadoughi University of Medical Sciences, Yazd, Iran.*; 3 *Zanjan University of Medical Sciences, Znjan, Iran.*

**Keywords:** Vaginal discharge, Nisseria gonorrhea, Chlamydia trachomatis, Prevalence

## Abstract

**Background:**

*Chlamydia trachomatis *and *Neisseria gonorrhoeae *are the most prevalent bacterial sexually transmitted diseases in women.

**Objective:**

The purpose of this study was to investigate the prevalence of gonorrheal and chlamydial infections and determination of related risk factors in married women with vaginal discharge attending gynecological outpatient department (OPD) in Zanjan in 2013-2014.

**Materials and Methods:**

In this cross sectional study, 100 married women aged 18-49 years with vaginal discharge were evaluated for signs and symptoms of gonococcal and chlamydial infections. Then cervical discharge samples and blood samples were collected from each subject for the detection of *Nisseria gonorrhea* and *Chlamydia trachomatis* by bacterial culture and serological tests, respectively.

**Results:**

The overall prevalence of *Chlamydia trachomatis* and *Nisseria* *gonorrhoeae* were 16% and 4%, respectively. There was no significant relationship between the contraception methods, previous history of vaginal infections, previous history of urinary tract infections, number of coitus per week and self-reported symptoms (itching, burning, abdominal pain) with prevalence of *Nisseria* *gonorrhoeae* and *Chlamydia trachomatis*.

**Conclusion:**

According to our results, the prevalence of gonococci infection in Zanjan was remarkable and relatively was higher than other parts of Iran, therefore it is necessary to put emphasis on education and further preventive and therapeutic programs.

## Introduction

Sexually transmitted infections (STIs) are major public health concern worldwide, especially in developing countries due to limited resources and facilities to diagnose and treat them (1). And each year globally occurred around 500 million new curable STIs (2). According report of WHO in 2012, the estimated global prevalence of chlamydia and gonorrhea was 4.2% and 0.8% respectively (3). 

Epithelial cells of the genital tract can infected through *chlamydia trachomatis *as an obligate intracellular pathogen in infected women. Most of infected women have no symptoms and clear from infection spontaneously, but persistent infection can spread to their upper genital tract (4).*Chlamydia trachomatis *causes long term complications such as pelvic inflammatory disease, chronic pelvic pain, ectopic pregnancy, and infertility (4). Gonorrhea is an infection due to *Neisseria gonorrhoeae *and usually involves mucosa of the cervix, urethra, endocervix, fallopian tubes, rectum, and throat (5, 6). Generally clinical symptoms for *gonorrhoeae* are not different from genital chlamydial infection and clinically are indistinguishable (4).* Chlamydia trachomatis* and *Neisseria gonorrhoeae* are asymptomatic in the most of infected women (7). 

Prevalence of chlamydia and gonorrhea in the country is considerable, so that results of the national meta-analysis study showed that C. trachomatis has a high prevalence for women in Iran and ranged from 0 to 32.7% in different studies (8). Afrakhteh et al. showed that Neisseria gonorrhoeae was the fourth prevalent STI pathogens in females in Tehran with the prevalence rate equal 4.91% (9). Literatures shows that Being young, female gender, number of sexual partners considered as risk factors of these two infections (10-12). But there is not enough evidence in regard of association between type of partners, use of contraceptive methods, and age of partners at first intercourse and increase risk of chlamydial (13). 

Increasing the risk of persistent undiagnosed chlamydial and gonococcal infections may lead to complications such as pelvic inflammatory diseases, infertility, ectopic pregnancy, tubal factor infertility, chronic pelvic pain, cervical infection, premature delivery, and low birth weight (14-18). The prevention of the occurrence complications and curb the spread of infection in symptomatic and asymptomatic patients usually is depends on early and accurate diagnosis and also appropriate treatment of infection (19). Also early detection of these STIs because of their synergistic effect with HIV infection is extremely important (20).

For proposing the strategies to prevent and control these infections we needed to precise data collection focused on distribution of *Chlamydia trachomatis* and *Neisseria gonorrhoeae* and demographic characteristics (21). "While high STI prevalence indicates frequent risky sexual practice and a poor provision or uptake of services, low STI prevalence reflects the improvement in provision of care services or change in risky behaviors"(22). Based on this evidence, it is essential to carry out such studies. 

Therefore, the purpose of this study was to investigate the prevalence of *Chlamydia trachomatis* and *Neisseria gonorrhoeae*, and determination of related risk factors in married women with or without symptoms in Gynecological OPD in Zanjan in 2013-2014. 

## Materials and methods

In this Cross sectional (descriptive/analytic study), 100 married women, 18-49 year old who were suffering from vaginal discharge with or without symptoms (itching, irritation and abdominal pain) referred to the Gynecology Clinic at Ayatollah Mousavi Hospital in Zanjan, west of Iran, during 2013-2014, were included in the study.

Virgin and pregnant women, women at the time of menopause, history of chronic diseases such as diabetes, kidney transplantation, immune system disorders, corticosteroid use, females with history of using vaginal cream, vaginal douching and sexual intercourse in the last week, as well as using oral antibiotic during the past 48 hr were considered as exclusion criteria’s. 

Data were gathered by a checklist. The checklist used in this study was including: data on demographic characteristics, body mass index (BMI), educational level, occupation, pregnancy status, vaginal and genital tract infections, and contraceptive method. Also, their symptoms such as local itching, burning, dysuria, abdominal pain, abnormal vaginal discharge, the amount of discharge (low, high, normal), discharge color (white, green, and yellow), and the smell of the discharge were listed. 

Then cervical discharge and blood samples were collected from each participant for the detection of *Chlamydia trachomatis* and *Neisseria gonorrhoeae* by bacterial culture and serological tests, respectively. The endocervix was first cleaned with a sterile cotton swab to remove mucous and exudates then samples were obtained from endocervix by a sterile applicator. Two cervical swabs for detecting of *Neisseria gonorrhoeae* were collected from participants by a gynecologist. One cervical swab was taken to prepare the smear on a sterile glass slide, and the remaining swab sample was placed into a test tube. Once the samples were obtained, they were transported to Laboratory at Ayatollah Mousavi Hospital in Zanjan, Iran. 

For isolation and identification of Neisseria gonorrhoeae, cervical swab samples were directly inoculated in modified Thayer Martin Media (MTM) (MERCK Co., Germany) and they were maintained in 5% CO_2_ at 37^o^C. Isolates were identified as *Neisseria gonorrhoeae* on the basis of colony morphology, Gram staining, the oxidase, catalase and carbohydrate utilization tests. Gram staining was performed using Gram stain kit (Bahar Afshan Co., Iran). In this study the Enzyme Immune Assay (ELISA) method was used to assess chlamydial infection. 2 mL blood sample was taken from each subject and was transferred to the laboratory for separation of serum. Serum IgM and IgG antibodies against *Chlamydia* were measured by enzyme-linked immunosorbent assay (ELISA) method (NOVA TEC kit, Germany).

**Ethical consideration**

The study protocol was approved by the Ethics Committee of Zanjan University of Medical Sciences, Zanjan, Iran. A written informed consent was taken from all participants. 

**Statistical analysis**

The collected data were analyzed using the Statistical Package for Social Science (SPSS Inc., Chicago, version 11.5). Descriptive results were expressed as mean and standard deviation. Chi-square tests (*x*^2^), and Fisher exact test were used for analysis of categorical data. *P*<0.05 was considered statistically significant.

## Results

There were 100 women with vaginal discharge, with a mean±SD age of 33.05±7.97 years. BMI ranged from 18.37-37.17 kg/m^2^ )mean±SD=26.78±18.37(. Most women had been referred to the clinic in the second half of the menstrual cycle. As shown in table 1, only 10% of patients had higher education than diploma and 23% were illiterate. 17% of participants in the study did not have a history of pregnancy or childbirth. 61% of participants used withdrawal method of contraception. Abdominal pain was the common observed clinical symptom among them (62%).

The overall prevalence rate of Chlamydia trachomatis infection was 16% (16 women) in our participants. ELISA tests indicated that 16 women had positive antibodies’ level in their blood: 1 woman had anti-Chlamydia trachomatis IgM antibody, 12 had positive IgG antibody, and 3 women had both of them.* Neisseria gonorrhoeae* were detected in 4 (4%) participants. According to table II, there was no significant relationship between the contraception methods, history of vaginal infections, history of urinary tract infections, number of sexual intercourse weekly, and self-reported symptoms with the prevalence of *Neisseria gonorrhoeae* and *Chlamydia trachomatis (*p>0.05).

**Table I:** Frequency of studied variables in 100 women with vaginal discharge attending gynecological OPD in Zanjan

**Variables**			**n**	**%**
Education of women				
	Illiterate		23	23
	Under diploma		40	40
	Diploma and associate degree		27	27
	BA and higher		10	10
	Age group			
	<30 year		58	58
	≥ 30 year		42	42
Number of pregnancy				
	0		17	17
	1		25	25
	2		30	30
	3≤		28	28
Contraceptive method				
	Pill		15	15
	DMPA		4	4
	IUD		4	4
	Tubectomy or vasectomy		9	9
	Condoms		7	7
	Withdrawal method		61	61
Number of coitus weekly				
	1-2		64	64
	3-5		29	29
	≥ 5		7	7
The clinical symptoms observed				
	Itching		59	59
	Burning		47	47
	Abdominal pain		62	62
The amount of discharge				
	Low		40	40
	High		39	39
	Normal		21	21
Discharge color				
	White		49	49
	Yellow		47	47
	Green		4	4
Type of discharge				
	Foamy		4	4
	Clear		38	38
	Thick		38	38
	Cottage cheese		20	20
	Unpleasant smell of the discharge		61	61

BA: Bachelors academic degree,

DMPA: Depomedroxyprogesterone acetate

IUD: Intea uterin device for contraception

**Table II:** Relation between studied variables and prevalence of Neisseria gonorrhoeae and Chlamydia trachomatis

**Variables**		**Chlamydia negative**	**Chlamydia positive**	**p-value**	**Gonorrhoeae negative**	**Gonorrhoeae positive**	**p-value**
History of UTI							
	Yes	14 (16.7)	4 (25)	0.48	16 (16.7)	2 (50)	0.14
	No	70 (83.3)	12 (75)		80 (83.3)	2 (50)	
history of vaginal infections							
	Yes	58 (0.69)	10 (62.5)	0.77	65 (67.7)	3 (75)	1
	No	26 (31)	6 (37.5)		31 (32.3)	1 (25)	
Contraceptive method							
	Pill	13 (15.5)	2 (12.5)	0.98	14 (14.7)	1 (25)	0.31
	DMPA	3 (3.6)	1 (6.3)		4 (4.2)	0	
	IUD	4 (4.8)	0		3 (3.1)	1 (25)	
	Tubectomy or vasectomy	8 (9.5)	1 (6.3)		9 (9.4)	0	
	Condoms	6 (7.1)	1 (6.3)		7 (7.3)	0	
	Withdrawal method	50 (50.5)	11 (68.8)		59 (61.5)	2 (50)	
Number of coitus weekly							
	1-2	50 (59.5)	14 (87.5)	0.14	62 (64.5)	2 (50)	0.69
	3-5	27 (32.1)	2 (12.5)		27 (28.1)	2 (50)	
	≥ 5	7 (8.3)	0		7 (7.3)	0	
The clinical symptoms observed							
	Itching	46 (54.8)	13 (81.3)	0.56	58 (60.4)	1 (25)	0.3
	Burning	39 (46.4)	8 (50)	1	46 (47.9)	1 (25)	0.6
	Abdominal pain	54 (64.3)	8 (50)	0.4	59 (61.5)	3 (75)	0.9

## Discussion

This is a cross sectional study regarding investigate the prevalence of *Chlamydia trachomatis* and *Neisseria gonorrhoeae*, and determination of related risk factors in married women with vaginal discharge in Gynecological OPD in Zanjan. The overall prevalence of *gonorrhea* infection in this study was 4%. Our results were consistent with the incidence reported by Afrasiabi *et al* using culture techniques in Kashan, Iran and Akya *et al* using PCR techniques in Kermanshah, Iran for *Neisseria gonorrhoeae* infection (21, 23). 

In the present study, the prevalence of gonococci infection was relatively higher than some of national studies. For example in a study conducted by Haghighi *et al* in Sabzevar, Iran, the reported incidence of *Neisseria gonorrhoeae* was 1.25% (24). In another study were done by Bakhtiari *et al* in the north of Iran, the incidence of *Neisseria gonorrhoeae* was 0.2% (25). In one study in Zanjan in 2009, 308 vaginal samples from women were evaluated. They reported that the prevalence of *Neisseria gonorrhoeae* was 0.9% (26). Similar studies have had different results at the international level, for example consistent with our results in a study in health clinic attendees complaining of vaginal discharge in Bangladesh, the prevalence of *Neisseria gonorrhoeae* has been reported 3.8%, a higher rate of gonococcal prevalence (4.0%) was reported in women of Brazil (27, 28). 

Also one study in Turkey that used PCR reported a *Neisseria gonorrhoeae* infection incidence of 3.4%, which was relatively similar to our findings (29). While some studies reported lower rate than our study, the prevalence of *Neisseria gonorrhoeae* was 1.9% in the symptomatic population in Italy (30). Furthermore Farraj and his colleagues in Palestine used PCR to examine 213 endocervical samples and reported a *Neisseria gonorrhoeae* infection incidence of 1.4% (31). Or the prevalence rates of *Neisseria gonorrhoeae* in urine samples study of Ramos de Lima *et al* were 0.7% by PCR. In this Brazilian study because the type of specimen used for diagnosis, prevalence rate was lower than our study. Although nucleic acid amplification tests are highly appropriate for the detection of genital gonococcal infection, In addition PCR test for demonstration of *Neisseria gonorrhoeae* detection in urine samples has lower sensitivity (5). By contrast in Australia, baseline prevalence was 9.5% for *Neisseria gonorrhoeae, *which was higher than our result (32). “The reasons for discrepancy in gonorrhea rates are not well understand, while probably include differences in access to health services and utilization, sample size, geographic clustering of populations, other interrelated social and economic factors, and sexual partner choices along both socioeconomic and racial lines “(33). *Gonorrhea* is a major public health concern globally that requires immediate international public health resources and attention (34). 

while the most of symptomatic infected women were remained untreated because they did not seek care, and 50% of asymptomatic infected women were undetected and untreated (35). Thus, the control of this disease will require strategies such as screening and mass treatment, in addition to improved clinical services for patients with symptoms (36). The isolation of *Neisseria gonorrhoeae* from endocervical specimens by culture is the suggested method for isolation of gonorrhea in women (21).

The overall prevalence of chlamydia infection in this study was 16%. This result suggests a high frequency rate of *Chlamydia trachomatis* infection in Zanjan, Iran. In our study the detection rate of *Chlamydia trachomatis* was relatively higher than the results of the other studies in Iran. Akya *et al* reported a 3.1% incidence of *Chlamydia trachomatis* by PCR in Kermanshah, Iran (23). In the United States the incidence of *Chlamydia trachomatis* in young adults has been reported around 10%, which was lower than our findings (37). 

The prevalence of *Chlamydia trachomatis* in our study (16%) is higher than sexually active young Brazilian women (7.4% and 13.5%) that were examined in two separate study (38, 39), and also in similar studies in Greenland, USA, Norway, Canada and Uganda with the prevalence of chlamydia infection 7%, 8%, 7.2%, 9.3% and 6% respectively (40-44). However differences in the prevalence of *chlamydia* and *gonorrhea* in different studies may be due to the use of different diagnostic methods, differences in sample size and different socio-cultural conditions. 

In our study the prevalence of infection for both organisms was higher in women aged younger than 30 year (58% vs. 42%). Similarly, some of studies showed that the younger aged women are at higher risk for *Chlamydia trachomatis* and *Neisseria gonorrhoeae *(5, 21, 23). It is obvious that the younger aged women have higher sexual promiscuity therefore they are at higher risk for STD (45). According to findings of this study bacterial infection has higher rate among women with use of withdrawal method. These results are similar to the results reported by Yirenya-Tawiah *et al* and Akya *et al* (23, 46). As shown in the results of this study, only 7% of women have used barrier methods, it seems that the high prevalence of chlamydia and gonorrhea among women in this study partly can be explained through it.

Our findings did not show any relation between self-reported symptoms and positive labratoary tests for chlamydial and *gonorrhoeae*. These results was concordance with the study performed by Yirenya-Tawiah *et al* in Ghana, Nevertheless, they observed only a small percentage of the STI positive cases to report the presence of symptoms related to STIs (46). In a study was done in Batswana, many *Chlamydia trachomatis* and *Neisseria gonorrhoeae* infected individuals were asymptomatic (47). In another study by Akya *et al*, the difference in the rate of infection for both *Chlamydia trachomatis* and *Neisseria gonorrhoeae* among symptomatic and asymptomatic women was not statistically significant, indicating the silent infections of these bacteria among women (23). Consistent with our finding, Wasserheit *et al* in a meta-analysis study demonstrated that only 28% of women with *Chlamydia trachomatis* and *Neisseria gonorrhoeae* infection were reported to show symptom of vaginal discharge (48). Wasserheit *et al also found that in 92% of women with *vaginal discharge were not infected to gonococcal and chlamydial infection. She concludes that syndromic approach should not be used as a screening procedure for case finding of gonococcal and chlamydial (48).

**Limitations**

The study presents some limitations; clinical data such as sexual behavior and number of sexual partners was not consistently recorded and were thus not included into the study results. A number of patients were not working to obtain blood samples and leading to longer process of collecting samples. To identify high risk patients of transmission STD, we required a detailed biography about sexual relations, multiple sexual partners, type and history of marriage, that none of the patients cooperated to complete information. Also patients did not give us properly information about clinical signs of their intimate partner, these cases may be due to constraint issues related to face-to-face interviews. Future studies are needed to carefully record sexual partner and provide additional epidemiologic information.

## Conclusion

The results of this study revealed the prevalence of *Chlamydia trachomatis* and *Neisseria gonorrhoeae* in women suffering from vaginal discharge is relatively high. It may be because there is no clinic for sexually transmitted diseases in Zanjan, Iran and patients with urogenital infections referred to gynecology which can increase the positive results. According to results obtained through laboratory tests, the prevalence of gonococcal and chlamydial infection in Zanjan is relatively high, which makes it necessary to put emphasis on education and further preventive and therapeutic programs. It seems that the prevalence of asymptomatic chlamydial and *gonorrhoeae* infection in women to be high, therefore focus on clinic-based testing is inadequate.

## Note

***This article extracted from M.D. thesis. (Atefe Tirkan, Mahsa Kiani)***
